# A four phase development model for integrated care services in the Netherlands

**DOI:** 10.1186/1472-6963-9-42

**Published:** 2009-03-04

**Authors:** Mirella MN Minkman, Kees TB Ahaus, Robbert Huijsman

**Affiliations:** 1 Vilans, National Center of Excellence in Long-term care, Catharijnesingel 47, PO Box 8228, 3503 RE Utrecht, the Netherlands; 2 University of Groningen, Faculty of Economics and Business, Research Center on Healthcare Organization & Innovation, University Medical Center Groningenm, Landleven 5, 9747 AD Groningen, the Netherlands; 3 Erasmus University Rotterdam Institute of Health Policy and Management, PO Box 1738, 3000 DR Rotterdam, the Netherlands

## Abstract

**Background:**

Multidisciplinary and interorganizational arrangements for the delivery of coherent integrated care are being developed in a large number of countries. Although there are many integrated care programs worldwide, the process of developing these programs and interorganizational collaboration is described in the literature only to a limited extent. The purpose of this study is to explore how local integrated care services are developed in the Netherlands, and to conceptualize and operationalize a development model of integrated care.

**Methods:**

The research is based on an expert panel study followed by a two-part questionnaire, designed to identify the development process of integrated care. Essential elements of integrated care, which were developed in a previous Delphi and Concept Mapping Study, were analyzed in relation to development process of integrated care.

**Results:**

Integrated care development can be characterized by four developmental phases: the initiative and design phase; the experimental and execution phase; the expansion and monitoring phase; and the consolidation and transformation phase. Different elements of integrated care have been identified in the various developmental phases.

**Conclusion:**

The findings provide a descriptive model of the development process that integrated care services can undergo in the Netherlands. The findings have important implications for integrated care services, which can use the model as an instrument to reflect on their current practices. The model can be used to help to identify improvement areas in practice. The model provides a framework for developing evaluation designs for integrated care arrangements. Further research is recommended to test the developed model in practice and to add international experiences.

## Background

Integrated care programs are being developed in countries all over the world in order to reduce fragmentation in care and to improve clinical outcomes, quality of life, patient satisfaction, effectiveness (use of evidence-based guidelines) and efficiency or reduce costs [1,2]. Integrated care is defined as a coherent and co-ordinated set of services which are planned, managed and delivered to individual service users across a range of organizations and by a range of co-operating professionals and informal carers [3]. Developing integrated care services is complex. Arranging streamlined patient flows, establishing partnership relationships among health care organizations and linking planning and information systems are some examples of activities within these complex processes. Although there are many integrated care programs worldwide, the process of developing these programs and such interorganizational collaboration is described in the literature to only a limited extent [3-5]. In related areas, like the development of organizations, a body of literature is available. Interesting questions are therefore how the development process of these programs can take place in practice and what activities can characterize these developmental processes over time. We first review some of the main literature in three related areas: organizational development, the development of networks, and quality management models in health care based on assumptions concerning the development of organizations or networks to improve performance. We focus on how the development processes are described and with what characterizing features.

### Organizational development

Since the late 1960s there have been a number of publications about organizational development [6-12]. These authors suggest that the development and behavior of organizations can be predicted by means of organizational life-cycle models according to which changes in organizations follow a predictable pattern involving developmental stages. Most authors suggest three to five sequential stages, sometimes in parallel with natural growth stages such as birth, youth and maturity. Greiner [7] developed one of the earliest models in the private sector and defined six phases of growth, each followed by a revolution or transitional phase arising from a major organizational problem. The sixth phase later added refers to extra-organizational solutions like alliances, networks or mergers of organizations. D'Aunno and Zuckermann [12] describe a four-phase life-cycle model for federations in health care. Federations are defined as interorganizational collaboration by at least two membership organizations, guided by a management group. Based on earlier life-cycle models, they define four phases: 'emergence of a coalition', 'transition to a federation', 'maturity of the federation' and 'critical crossroads'. For each stage they define two key factors and examples of tasks such as 'defining the goal of the coalition' in the first stage. Because empirical evidence for the model is lacking, the authors suggest testing some hypotheses. Although there may appear to be consensus about life-cycle thinking, Phelps [13] points out the limits of life-cycle models. According to Phelps there is an absence of consensus about the number of phases, phase characteristics and phase definitions. Moreover, the assumption that organizations do experience life cycles is based on literature that it is mainly conceptual and descriptive in nature. In addition, the parallel with linear growth stages is doubted, and an evolutional or a discontinuous perspective would appear more realistic [14]. Studies from the latter perspective are problem-oriented and define transitions between phases in terms of the dominant management problems to be addressed [15,16]. To summarize, there is a consensus in the literature that organizations change over time in response to important problems related to survival. Despite criticism, a large number of authors describe multiple phases of organizational development, but the phase characteristics and transitions from phase to phase differ widely. The underlying empirical evidence for most models is limited and growth models can best be used in conceptual discussions about organizational development or as descriptive devices to represent patterns that have emerged [17].

### Network organizations

A second related area is the development of networks. A network can be defined as more or less stable patterns of social relations among different actors (people, groups, organizations) who depend on each other to reach their goals without the existence of a dominant actor. Network relations imply that coordination among actors takes place on the basis of mutual benefit, reciprocity and trust [18]. There have been very few published reports evaluating ties among organizations in various types of network organizations in health care [5]. The limited evidence available on the effects on client outcomes are equivocal, with some finding no relationships and others finding support [19-21]. The logic underlying collaborative networks is however strong and compelling. Information-sharing within the network and organizational commitment to the network are of overriding importance. The complexity of this approach however, is that collaborating organizations often have different goals, funding streams and stakeholders, meaning that integration is not easily achieved in practice [5]. This implies that the process of building a collaborative interorganizational network can be difficult; as new relationships develop and the attitude towards the process remains positive, the level of trust may even decline [5,22].

A study of various forms of network organizations in the business sector identified trust and equity as important issues in the development process of an interorganizational relationship [14]. The three stages of 'negotiations of joint expectations by formal bargaining and informal sense making', 'commitments for future actions' and 'execution of commitments' repeat and overlap one another and have a duration depending on the reliance on trust and role relationships. From a developmental perspective one conclusion is that network organizations will continually shape and restructure over time as a result of the actions and interpretations of the parties involved [14]. The limited studies and evidence available stress the need for more knowledge about these processes.

### Quality improvement models

A range of quality management models is available for increasing the performance of health care organizations. Two models used in health care with assumptions concerning the process of development or levels of implementation are the Chronic Care Model (CCM) and the European Foundation Quality Management Excellence model (EFQM model) [23-26]. The CCM defines four levels, named 'A till D', in which level D describes components of the model in a limited implementation stage and level A describes the most developed stage. For example 'organizational goals of chronic care' do not exist or are limited to one condition at level D, but are measurable, reviewed routinely and incorporated at level A. The levels are described for providers to assess their situation and identify areas for improvement. The Dutch version of the EFQM model describes five phases of organizational growth, namely 'activity-oriented', 'process-oriented', 'system-oriented' 'chain-oriented' and 'transformation-oriented'. A complementary Dutch EFQM model for chain management uses the same phases for the development of interorganizational collaboration [27]. This expert-based model is of interest for integrated care for its dominant focus on interorganizational collaboration to optimize the total results of the care chain. However, the model's components in each phase are described at a generic level only and are not specified for health care. To summarize, both the CCM and EFQM model suggest phases or implementation stages. The description of phases is lacking (CCM) or is generic and not health-care specific (EFQM). The empirical evidence underlying the models is based on expert opinion.

From a review of the literature concerning organizational development, network organizations and quality improvement models, it appears that developmental processes are frequently described in the form of multiple stages or phases with different characteristics. How phases are defined and characterized differs and the evidence levels are merely based on expert opinion. It remains unclear if integrated care programs develop similarly. We therefore conducted this two-step study to answer the following research questions: How can the development process of integrated care programs be described and characterized? What essential elements of integrated care are important in each part of the development process?

## Methods

**Figure 1:**
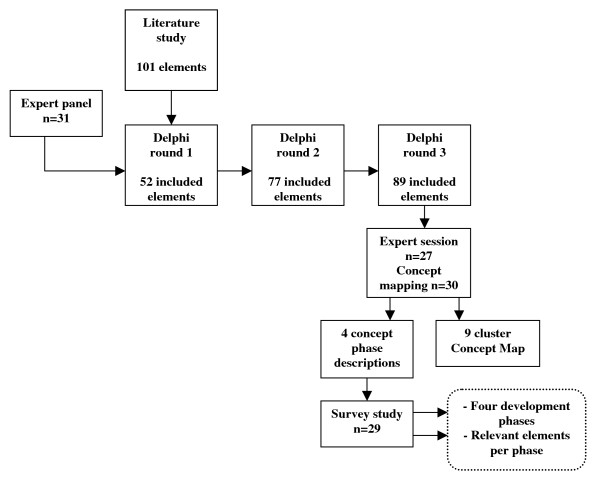
**Study design**.

A two-step study design was used, see figure 1. To research what elements of integrated care are important in the developmental process, a consistent set of essential elements of integrated care is needed. The development of this set was the first step of our study. In the second step, which is the focus of this article, these elements are further researched in relation to the developmental process of integrated care. Because of the explorative nature of our research, we used multiple methods and qualitative and quantitative analyses to generate an empirical conceptual model of a complex process [28].

### Elements of integrated care (part one)

To assess essential elements of integrated care, a pre-study involving a literature study, a Delphi study and Concept Mapping was conducted [29]. A structured multiple-source literature study (reviews, articles, theses, evaluation reports, quality management models) resulted in a list of 101 elements of integrated care. An element of integrated care is defined as an activity focusing on the development (realization, improvement, innovation or sustainability) of integrated care, based on the quality continuum of Feussner et al. [30]. In order to improve, complete and restrict the list of elements, a systematic Delphi study was carried out with a panel of 31 experts. Experts met the following criteria: multiple years of experience with integrated care, experience with multiple and different patient groups or integrated care settings, and expert knowledge based on research, implementation projects or practical experience. All experts approved their participation by personal and e-mail confirmation for all parts of the study. No ethical approval was required because because this research did not include patient but professional experts.

During three anonymous Delphi rounds each expert rated all elements on a four-category Likert scale with the following response categories: not important, moderately important, important or very important. Experts could make suggestions for reformulation of each element and could add new elements. After each round, elements were included (if > 80% scored the element as important or very important) or excluded (if > 50% scored the element as not important or moderately important). These cut-off points were set in consultation with methodologists. New elements, reformulated elements and elements that were neither included nor excluded were presented in the next round. If suggestions for reformulation were made, they were analyzed individually by the researchers and reformulated on the basis of consensus. This systematic procedure resulted in a list of 89 elements of integrated care. All 31 experts completed all three Delphi rounds, which resulted in a 100% response score of this study.

To further analyze the list of elements, Concept Mapping was applied. During a session with the expert panel, 30 of the experts individually clustered the elements by means of a computerized groupware system. The data generated by the experts were stored in a database and used for the statistical procedure, which was carried out by the computer program ARIADNE version 2.0 [30]. Firstly, a point map was calculated by using multidimensional scaling [31]. The scaling procedure positioned each element on a two-dimensional map with four poles. Secondly, the coordinates of the point map were used in order to conduct hierarchical cluster analyses. After reviewing several cluster maps by following the advised procedure [32,33], a nine-cluster solution best fitted the conceptual framework. In the next step nine subgroups of experts each analyzed one cluster with its elements and formulated a cluster label and a cluster description. The labels of the clusters were defined as: 'Quality care', 'Performance management', 'Interprofessional teamwork', 'Delivery system', 'Roles and tasks', 'Patient-centeredness', 'Commitment', 'Transparent entrepreneurship' and 'Result-focused learning' [29].

### Development process of integrated care (part two)

#### Expert session

In the second part of this study we used the expert panel session and invited the same 31 Delphi panel experts to participate in a subsequent questionnaire study. A study protocol for the expert session was followed. All experts approved again their participation by e-mail confirmation for this second part of the study.

After the cluster exercise at the expert session, a three-step approach was used. Firstly, the facilitator introduced the question as to how the developmental process of integrated care could be characterized. After a plenary discussion, resulting in consensus that different developmental phases were recognized in practice, the experts were divided into nine groups. The groups were organized according to the panel characteristics of 'background' and 'years of experience' to balance the expert characteristics between the subgroups. Each group discussed whether, how many, and with what characterizing features developmental phases of integrated care are recognized in practice. Each group made notes on a prestructured sheet. The subgroup discussions were observed by the five members of the research team. In the third step, all subgroup notes were taken by the researchers and the results were presented in plenary and discussed. Both plenary discussions were taped and two researchers independently made notes of the discussion.

#### Questionnaire

The results of the expert panel session were analyzed by means of mutual comparisons of subgroup phase descriptions on the sheets. Apart from the subgroup analyses, the transcription and notes of the taped discussions were analyzed by two researchers. Based on these analyses, a concept description of a four-phase model was constructed.

To further develop and member-check the concept model with the panel, a two-part Excel based questionnaire was developed and e-mailed to the experts. In the first part, the phase descriptions were presented and the experts were asked whether phase descriptions were recognized in practice (yes, partly, or no). The experts could make comments or suggest reformulations. If suggestions for reformulation were made, these were analyzed individually by three researchers and reformulated on the basis of consensus. In the second part of the questionnaire, each expert individually reviewed the 89 elements of integrated care from the pre-study in relation to the four phases. Firstly, they were asked to mark in which of the four phases they felt the element was most relevant (scoring a double X, the total maximum score per phase is 2581). Secondly, for each element they marked whether they were also relevant in any of the other three phases (scoring a single X). For further analyses, a weight of three was assigned to each double X score and one to each single X score. The rationale for this non-linear scoring procedure is as follows. There was substantial consensus in the panel that the development phases are connected and that there are no (strict) boundaries between the phases. Elements can be relevant in multiple phases. Therefore, a forced choice scoring (only one score per element) was not useful. After consulting methodologists, assigning the weights of 3 and 1 seemed to be the most unambiguous scores. Other scoring methods have been explored and are reported in the result section. Descriptive statistics and frequency analyses were further used to analyze the results.

## Results

### Expert session

Of the 31 experts, 27 attended the expert-session and 29 responded to the questionnaire (response 94%). The characteristics of the experts are reported in table 1. In the plenary discussion, the experts reached consensus that the development process could be characterized by multiple distinguishable phases. The nine subgroups defined phases which ranged between three (four groups), four (three groups) and five (two groups) phases. The following plenary discussion resulted in a consensus that a four-phase description appeared to cover all the named aspects best. The phases were called 'initiative and design phase' (phase 1), 'experimental and execution phase' (phase 2), 'expansion and monitoring phase' (phase 3) and 'consolidation and transformation phase' (phase 4). Further analyses based on mutual comparisons of the subgroup sheets resulted in a compact description for each of the four phases including three key words. The results were used as input for the questionnaire research.

**Table 1:** Respondent characteristics

**Characteristics**	**Category**	**Expert group** **N = 29**
Gender	Male	41%
	Female	59%

Age (years)	Min – Max	27 – 63
	Average (sd)	44.69 (9.39)
	< 40	28%
	40 – 50	48%
	> 50	24%

Years of experience	Min – Max	2 – 22
	Average (sd)	8.36 (4.80)
	< 5	21%
	5 – 10	55%
	> 10	24%

Source of expertise	Research	14%
	Research & practice	3%
	Implementation programs	28%
	Research & impl. programs	28%
	Practice & impl. programs	28%

Dominant background	Professional	52%
	Organizational/health sciences	48%

### Questionnaire: Phase descriptions

Analyses of the questionnaire results showed a high percentage of confirmation of the phases described. The description of phase three was mostly fully recognized (86.2% n = 25), followed by phase four (82.8% n = 24), one (79.3% n = 23) and two (69.0% n = 20). The percentages of experts that partially recognized the description were 20.7% (n = 6) for phase one, 31.0% for phase two (n = 9), and 13.8% for both phase three and four (n = 4). Only one expert stated not to recognize one phase (phase four). The results did not show contradictory suggestions of the experts, so consensus on all remarks was achieved in the research team. Remarks concerning phase one (the initiative and design phase) were that not only a mutual problem but also a chance or already existing collaboration can lead to the start of an integrated care program. Next to defining the targeted patient population, the supply chain is defined and the collaboration could result in a signed-up agreement between parties in the care chain. Refinements of phase two, the experimental and execution phase, were the allotment of coordinating roles and the clarification of roles within the care chain. Another addition was mechanisms of knowledge transfer within the integrated care. The panel comments on phase three, the expansion and monitoring phase, were limited and led only to the inclusion of innovation among the key words. In the fourth phase, the consolidation and transformation phase, inclusion of information feedback loops and the continuous assessment of client and stakeholder needs were added. Further analyses of the experts' remarks resulted in the following phase descriptions and key words:

#### PHASE 1 Initiative and design phase

The collaboration between health care providers has been intensified or started up. The starting point is a common problem or chance occurrence, or builds on current cooperation among care professionals. There is a sense of urgency and there are possibilities for working on these challenges in collaboration. The targeted patient group, the care chain and care process have been defined, as also the needs of patients and stakeholders. The level of ambitions, motivation and leadership determine the progress achieved. A multidisciplinary team designs an experiment or project to execute the present ideas. The collaboration can be signed up in an agreement among care partners.

Key words: Exploring possibilities/impossibilities, ambitions and chances, (project) design and collaboration agreements.

#### PHASE 2 Experimental and execution phase

New initiatives or projects are being executed in the care chain. The aims, content, roles, and tasks in the care chain have been clarified and written down in care pathways and protocols. There is coordination on the level of the care chain by for instance installing coordinators or setting up meetings. Information about patient groups, working procedures or professional knowledge is exchanged. There are experiments within the collaboration, results are evaluated to learn from and reflect on. Preconditions for projects have been considered and boundary conditions have been solved by collaborative means or agreements among care providers.

Key words: Writing down aims and content of the collaboration, coordination at care chain level, experimenting and reflecting.

#### PHASE 3 Expansion and monitoring phase

Projects have been expanded or integrated in integrated care programs. Agreements on the content, tasks and roles within the care chain are clear and signed up. Collaboration is no longer on an informal basis. Results are systematically monitored and improvement areas identified. The targeted population has been surveyed. More collaborative initiatives emerge such as mutual education programs. There is a continuous commitment to the ambition of the integrated care program. Interorganizational barriers and fragmented financial structures are on the agenda of care partners.

Key words: Further development and maturity, monitoring and improving results, new questions and innovation.

#### PHASE 4 Consolidation and transformation phase

The integrated care program is the regular way of working and providing care. Coordination at care chain level is operational; information is being shared, transferred and fed back. A monitoring system periodically shows if results are sustained, what specific improvement possibilities have been identified and to what extent patient needs have been met. The program builds further on successful results. Organizational structures transform or are newly designed around the integrated care program. Financial agreements are arranged with financers by means of integral contracts covering the care chain as a whole. Partners in the care chain explore new options for collaboration in the external environment with other partners.

Key words: Continuous improvement, new ambitions, structures fitting the integrated care program (organizational structures, integral financing).

### Questionnaire: Elements of integrated care

Twenty-nine experts each rated the 89 elements (response 94%). In total 77 out of the 89 elements were rated by at least one expert as mostly relevant in all four phases, 11 elements were rated in three phases as mostly relevant and one element was rated in two phases. All of the 89 elements were scored as relevant in the four phases by at least one expert. Only two elements were not scored as relevant in one phase by the experts. The total results are presented in additional file 1.

Of the total numbers of 'most relevant' scores, 812 were scored in phase two, 781 in phase three, 675 in phase one and 313 in phase four. The most scores of 'also relevant' were scored in phase four (1072), the least in phase one (428), and 783 and 945 scores in phases two and three. By assigning the weights as described in the methods section, the top 10 elements of every phase have been calculated (see tables 2, 3, 4 &5). Other scoring methods have been explored (e.g. assigning weights of 1 and 5 or 0 and 1), but gave no significant differences in the top ten elements of all four phases.

**Table 2:** PHASE 1. Initiative and design phase

***Rank***	Weight*	***Element description***
1	65.83%	Defining the ambitions and aims of the collaboration in the care chain

2	65.49%	Defining the targeted client group

3	52.46%	Defining and assessing the characteristics of the collaboratively delivered care

4	46.15%	Assuring the leadership commitment of the partners involved in the care chain

5	45.08%	Committing to a joint responsibility for the final goals and results to be achieved

6	42.37%	Establishing dependencies among care partners

7	41.13%	Describing the tasks and authorities of leaders, coordinators and advisory boards in the care chain

8	40.87%	Reaching agreements on referrals and transfer of clients through the care chain

9	40.83%	Signing collaboration agreements among care partners

10	40.34%	Reaching agreements on procedures for the exchange of client information

**Table 3:** PHASE 2. Experimental and execution phase

***Rank***	Weight*	***Element description***
1	52.76%	Realizing direct contact among professionals in the care chain

2	48.36%	Using shared client treatment and care plans

3	47.90%	Bringing specialized nurses into action through the care chain

4	46.92%	Achieving adjustments among care partners by means of direct contact

5	45.11%	Using evidence-based guidelines and standards

6	44.80%	Monitoring successes and results during the development of the integrated care chain

7	44.35%	Reaching agreements among care partners on discharge planning

8	43.85%	Working in multidisciplinary teams

9	42.86%	Ensuring that professionals in the care chain are informed of each other's expertise and tasks

10	42.52%	Gathering data on client logistics (e.g. volumes, waiting periods and throughput times) in the care chain

**Table 4:** PHASE 3. Expansion and monitoring phase

***Rank***	Weight*	***Element description***
1	50.41%	Using a systematic procedure for the evaluation of agreements, approaches and results

2	49.14%	Flexible adjustment of integrated care corresponding to individual clients' needs

3	47.20%	Monitoring and analyzing mistakes/near mistakes in the care chain

4	46.67%	Reaching agreements on introducing and integrating new partners in the care chain

5	46.40%	Using collaborative education programs and learning environments for the professionals of care partners

6	45.38%	Involving client representatives in improvement projects in the care chain

7	45.30%	Designing care for clients with multi- or co-morbidities

8	44.35%	Collaborative learning in the care chain in order to innovate integrated care

9	43.97%	Developing connections between databases of partners in the care chain

10	43.90%	Making transparent the effects of the collaboration on the production of the care partners

**Table 5:** PHASE 4. Consolidation and transformation phase

***Rank***	Weight*	***Element description***
1	40.18%	Offering a single collaborative financial contract to financing parties by the collective of care partners

2	39.17%	Linking consequences to the achievement of agreed goals

3	39.02%	Integrating incentives for rewarding the achievement of quality targets

4	29.77%	Structural meetings with external parties such as insurers, local governments and inspectorates

5	29.69%	Sharing knowledge among care partners about effectively organizing sustainable integrated care

6	28.80%	Using collaborative education programs and learning environments for the professionals of care partners

7	28.00%	Monitoring and analyzing mistakes/near mistakes in the care chain

8	27.27%	Developing care programs for relevant client subgroups

9	27.27%	Reaching agreements about letting go care partner domains

10	27.20%	Reaching agreements on the financial budget for integrated care

* Percentage of the total element score appointed in this phase (most important weight 3, also important weight 1)

The questionnaire results show that the description of phase four is highly confirmed (82.8%, n = 24), but the least numbers of elements are assigned to this phase as 'most relevant' whereas the most 'also relevant' scores are given in this phase. Experts remarked in the discussion that the fourth phase is recognized, but sometimes also partially a desired phase for the near future.

## Discussion

### Study reflections

Our explorative study resulted in a four-phase model that describes developmental phases of integrated care programs in the Netherlands. The phase descriptions were individually member-checked and confirmed by an expert panel. The dedication of the experts during the total study was remarkable and resulted in nearly perfect response rates, which indicates the study relevance. Our findings regarding the number of phases corresponds with the review of Phelps et al. [13]. In their review of 33 life-cycle models for organizations, about 70% of the models describe three to five phases, with the most (nine models) describing four phases. Quinn and Cameron [10] also composed a four-phase model based on their analyses of nine life-cycle models and concluded that common stages of development can be identified. As expressed in the plenary and subgroup discussions, the phases are meant to describe and characterize, not to prescribe or predict. The phases give an overview of commonly acknowledged processes or activities without any judgement about what phase is best when. This is a difference from some of the life-cycle models in the international literature, as these models sometimes assume 'predictable patterns' that organizations will or should follow. Interestingly, the experts do not define a phase of decline or termination of the development process, whereas in practice programs also sometimes end.

The similarities between the qualitative descriptions of the phases and the top ten elements in each phase (tables 2, 3, 4 &5) are evident. Like in the description of the first phase, elements that focus on defining the domain of integrated care, operational interorganizational processes (such as arranging patient transfers) and commitment are stressed as being the most important to realize. In the second phase too, elements that arrange coordination and streamline care processes are to be found in both study results. However, direct contact (as the most important element in phase two) appears more implicit in the description, but is necessary for the exchange of working procedures or professional knowledge. For the third and fourth phases the overlap is also clear, whereas the elements sometimes point out more specific examples (like 'analyzing near mistakes') of more generic formulations in the phase descriptions (like 'systematically monitored results').

### Study findings related to the main literature

Regarding the related literature, there is some overlap with organizational life-cycle models such as Quinn and Cameron's [10] four-phase model. As in our model, their first phase is the entrepreneurial stage in which lots of ideas, entrepreneurial activities and little planning and control are present. Their fourth 'elaboration of structure' stage contains domain expansion, renewal and changing structures which are comparable with activities within our consolidation and transformation phase. However, their second phase focusses on collectivity and their third phase on formalization and control. Whereas in our model the intensity of control and coordination also increases in each phase, the structures are not stable or focused on conservation. Like in the literature on networks, the parties in the collaboration cause the integrated care program to shape and restructure over time, and to expand, innovate and transform [14].

When looking at the characteristics of the phases, the intensity of collaboration and the nature of the activities show different emphases in each phase. The levels of integration as defined by Leutz [34] – linking, coordinating and full integration – are mirrored in the descriptions. In the 'initiative and design' phase, the linking of providers, through cooperation, the sharing of information and definition of responsibilities for each service without shifting costs and responsibilities is present. In the second and third phases, coordination is the dominant level and explicit structures and managers are installed in order to coordinate benefits and care across the care program. As in the case of Leutz, in our second and third phases the integrated care operates largely through the separate structures of the current systems. Leutz's third level of 'full integration' is mirrored in our fourth phase description where new programs or resources from multiple systems are pooled and structures transform.

In relation to the frequently used CCM and EFQM, there are some parallels with (in particular) the EFQM model. The EFQM defines five phases and appears to point out a more stepwise and rational model, where this study's model also emphasizes aspects such as commitment, contact, opportunities, and experiments. The importance of trust, commitment, and equity as mentioned in the literature on networks appears to contribute to the interorganizational collaboration in integrated care.

The Chronic Care Model defines four stages of development, but the stages themselves are not described. A difference is that the elements within the CCM differ in intensity or presentation per phase, but show an increased level from phase D to phase A. In our model, a number of elements are merely phase-specific and are not all that relevant in others. Each upcoming phase is not (only) a step further in development, but can also have new and phase-specific characteristics.

### Research limitations and implications

The systematic Delphi approach, which had as its starting point a systematic literature study combined with the strictly followed procedures of Concept Mapping and standardized computer-supported statistical analyses, contributes to the internal validity of this study. Using a protocol for the expert session and executing analyses of the results by multiple researchers also contribute to this. Although a committed expert panel with extensive experience in integrated care was involved in this study, our explorative study has the limitation that it uses the expert opinion of a panel of Dutch experts. Contextual factors such as the type of health care system, social values, health reform, the history of quality and the language and politics of quality will have influenced the results [35]. However, we think that for multiple reasons the study is of value for many readers in other countries. Firstly, in our literature study we included international literature which was the input for the Delphi study and the elements of the model. Secondly, the focus of the model is on integration processes. As described by Nies and Bergman [2] and Van Raak et al. [3], in a lot of countries there are separate sectors for acute care, long term care and social care. A mutual problem in these countries is how to integrate care processes. The Dutch health care system is a complex social insurance-based one with multiple components and a clear split between acute health care and long-term and social care [3]. For a large number of patients, health care professionals from all three sectors are involved. Within this complex system, contradictory impulses are send out by the Dutch health care policy makers. On the one hand integrated care is stimulated, but at the same time competition is stimulated and new financial structures do not facilitate integrated care. This complex and fragmented situation assumes that the study results will be of value for other systems that also experience a lot of fragmentation. Lastly, our expert panel consisted partly of experienced researchers in integrated care, who also have participated in international studies before. However, Dutch contextual factors may have played a role in our study. Therefore a suggestion for further research is to expand this study to other countries.

### Practical implications and further research

The development model can be used as an assessment and discussion tool in integrated care practice. Managers and professionals can use the model to reflect on the development of their practice, to discuss which elements are or are not present and to identify improvement suggestions. Together with the nine clusters of integrated care and the concept map developed in the pre-study [29], a rich model for assessing and improving integrated care practices has been developed. A suggestion for further research is to improve the external validity by replication of the study in other countries and healthcare systems. Another suggestion for further research is to use the model as a framework for evaluation designs to assess the development of integrated care programs. The relationship between the developmental process and outcomes of care is another suggestion for further study. Interesting questions are whether different developmental phases relate to different outcomes, or what characterizes integrated care programs with the best performance. Lastly, it may be assumed that managers and professionals will need different competences in the different phases. Research providing a further insight into each developmental phase is therefore recommended.

## Conclusion

This study provides a descriptive model of the development process that integrated care services can undergo in the Netherlands. Integrated care development can be characterized by four developmental phases: the initiative and design phase; the experimental and execution phase; the expansion and monitoring phase; and the consolidation and transformation phase. Different elements of integrated care have been identified in the various developmental phases. The findings have important implications for integrated care services, which can use the model as an instrument to reflect on their current practices and help to identify improvement areas. The model provides a framework for developing evaluation designs for integrated care arrangements. To conclude, the limited literature and evidence about the developmental process of integrated care programs emphasize the relevance of this explorative study. The wide-ranging attention towards integrating care and developing integrated care arrangements in developed countries underlines the need for further research on this topic by means of replicating or expanding this study.

## Competing interests

The authors declare that they have no competing interests.

## Authors' contributions

MM analysed the data. MM, RH and KA interpreted the data. All authors conceived and designed the study, drafted the manuscript and approved the final version.

## Funding

None

## Pre-publication history

The pre-publication history for this paper can be accessed here:



## Supplementary Material

Additional file 1 Relevance of elements of integrated care per development phase. The data provided represent the percentages of experts that indicate each element as 'most relevant' or also relevant (in brackets) for every development phase. The elements are presented per cluster for each of the nine clusters of the model. Click here for file
