# Supplementary material for: A four phase development model for integrated care services in the Netherlands
Source: BMC Health Serv Res. 2009 Mar 4;9:42. doi: 10.1186/1472-6963-9-42 (PMC2660899; doi:10.1186/1472-6963-9-42)
Supplement: Additional file 1 — Relevance of elements of integrated care per development phase. The data provided represent the percentages of experts that indicate each element as 'most relevant' or also relevant (in brackets) for every development phase. The elements are presented per cluster for each of the nine clusters of the model. [file 1472-6963-9-42-S1.doc]

## Additional files

#### Additional file 1: Relevance of elements of integrated care per development phase

| **Cluster 1. Patient-centeredness**  This cluster is about developing integrated care and information flows tailored to specific patient groups/subgroups. Elements focus on integrated patient and care-process support information such as front offices, self-management support or information systems, and delivering care tailored to individual needs (e.g. multi-morbidity).   | **% most relevant (also relevant). N=29** | | | |  | | --- | --- | --- | --- | --- | | Phase 1 | **Phase 2** | **Phase 3** | **Phase 4** | **Element description** | | 20.7 (13.8) | 41.4 (34.5) | 20.7 (55.2) | 17.2 (48.3) | Providing understandable and client-centred information | | 6.9 (17.2) | 51.7 (27.6) | 24.1 (51.7) | 17.2 (37.9) | Collaboratively offering client information from the care partners | | 13.8 (10.3) | 17.2 (37.9) | 55.2 (17.2) | 13.8 (37.9) | Designing care for clients with multi- or co-morbidities | | 3.4 (13.8) | 51.7 (20.7) | 24.1 (51.7) | 20.7 (44.8) | Using self-management support methods as a part of integrated care | | 20.7 (13.8) | 24.1 (44.8) | 41.4 (31.0) | 13.8 (48.3) | Implementing care process-supporting clinical information systems | | 0 (17.2) | 20.7 (27.6) | 58.6 (20.7) | 20.7 (34.5) | Flexible adjustment of integrated care corresponding to individual clients’ needs | | 20.7 (13.8) | 31.0 (27.6) | 37.9 (31.0) | 10.3 (27.6) | Developing a front office: single entry point for client information | | 17.2 (6.9) | 31.0 (27.6) | 34.5 (41.4) | 17.2 (41.4) | Using a protocol for the systematic follow-up of clients | | 27.6 (6.9) | 6.9 (41.4) | 37.9 (37.9) | 27.6 (31.0) | Developing care programs for relevant client subgroups | |
| --- | --- | --- | --- | --- | --- | --- | --- | --- | --- | --- | --- | --- | --- | --- | --- | --- | --- | --- | --- | --- | --- | --- | --- | --- | --- | --- | --- | --- | --- | --- | --- | --- | --- | --- | --- | --- | --- | --- | --- | --- | --- | --- | --- | --- | --- | --- | --- | --- | --- | --- | --- | --- | --- | --- | --- |
| **Cluster 2. Delivery system**  Chain and client logistics, coordination mechanisms and procedures for streamlining the care process for the whole care chain is the main focus of this cluster. The reaching of agreements (e.g. logistics, sharing expertise), procedures (e.g. information exchange) or tools (e.g. care plans) in the care chain that are necessary from the client’s initial entry into the care chain until the final contact are reflected in this cluster.   | Phase 1 | | **Phase 2** | | **Phase 3** | | **Phase 4** | | **Element description** | | --- | --- | --- | --- | --- | --- | --- | --- | --- | | 51.7 (6.9) | | 34.5 (34.5) | | 10.3 (31.0) | | 3.4 (24.1) | | Reaching agreements on referrals and transfer of clients through the care chain | | 41.4 (13.8) | | 37.9 (20.7) | | 13.8 (34.5) | | 6.9 (31.0) | | Reaching agreements on procedures for information exchange (content, method, responsibilities) | | 13.8 (17.2) | | 27.6 (31.0) | | 34.5 (34.5) | | 24.1 (41.4) | | Using a single client-monitoring record accessible for all care partners | | 51.7 (10.3) | | 34.5 (27.6) | | 10.3 (34.5) | | 3.4 (37.9) | | Reaching agreements on procedures for the exchange of client information | | 13.8 (6.9) | 13.8 (27.6) | | 51.7 (20.7) | | 20.7 (44.8) | | Developing connections between databases of partners in the care chain | | | 10.3 (10.3) | 31.0 (24.1) | | 37.9 (41.4) | | 20.7 (37.9) | | Offering case management for clients with complex needs | | | 37.9 (3.4) | 27.6 (37.9) | | 27.6 (34.5) | | 6.9 (31.0) | | Reaching agreements on chain logistics (e.g. waiting periods and throughput times) | | | 10.3 (17.2) | 62.1 (17.2) | | 13.8 (48.3) | | 13.8 (37.9) | | Using shared client treatment and care plans | | | 24.1 (10.3) | 41.4 (17.2) | | 20.7 (44.8) | | 13.8 (34.5) | | Using uniform client-identification numbers within the care chain | | | 27.6 (13.8) | | 37.9 (34.5) | | 27.6 (34.5) | | 6.9 (34.5) | | Reaching agreements among care partners on the consultation of experts and professionals | | 27.6 (13.8) | | 41.4 (31.0) | | 20.7 (31.0) | | 10.3 (27.6) | | Reaching agreements among care partners on managing client preferences | | 31.0 (20.7) | | 44.8 (20.7) | | 20.7 (31.0) | | 3.4 (24.1) | | Reaching agreements among care partners on scheduling client examinations and treatment | | 27.6 (13.8) | | 51.7 (20.7) | | 17.2 (37.9) | | 3.4 (24.1) | | Reaching agreements among care partners on discharge planning | | 34.5 (13.8) | | 41.4 (20.7) | | 13.8 (27.6) | | 10.3 (17.2) | | Developing criteria for the inclusion and throughput of clients in the care chain | | 20.7 (13.8) | | 31.0 (31.0) | | 41.4 (20.7) | | 6.9 (24.1) | | Reaching agreements among care partners on providing care to waiting-list clients | | 17.2 (17.2) | | 62.1 (10.3) | | 13.8 (44.8) | | 6.9 (37.9) | | Bringing specialized nurses into action through the care chain | | 24.1 (24.1) | | 37.9 (17.2) | | 20.7 (20.7) | | 17.2 (27.6) | | Reaching agreements on linking clients to outside resources or community care partners | | 34.5 (3.4) | | 44.8 (24.1) | | 20.7 (27.6) | | 0.0 (27.6) | | Developing criteria for assessing clients’ urgency |   **Cluster 3. Performance management**  Measurement and analyses of the results of the care delivered in the care chain is the central theme of this cluster. Elements address performance targets at all levels, monitored by the standardized use of indicators. Indicators address client outcomes, client judgments, organizational outcomes and financial performance data. Mistake/near-mistake analysis, feedback mechanisms and improvement teams are also used to improve and manage the level of performance.   | Phase 1 | | **Phase 2** | | **Phase 3** | | **Phase 4** | | **Element description** | | --- | --- | --- | --- | --- | --- | --- | --- | --- | | 27.6 (20.7) | | 17.2(34.5) | | 41.4 (27.6) | | 13.8 (34.5) | | Defining performance indicators to evaluate the results of the integrated care delivered | | 6.9 (13.8) | | 41.4 (27.6) | | 41.4 (34.5) | | 10.3 (58.6) | | Providing feedback to care partners on transfers | | 10.3 (10.3) | | 44.8 (34.5) | | 41.4 (41.4) | | 3.4 (44.8) | | Gathering client-related performance data (health status, quality of life) | | 17.2 (10.3) | | 51.7 (31.0) | | 27.6 (44.8) | | 3.4 (51.7) | | Gathering data on client logistics (e.g. volumes, waiting periods and throughput times) in the care chain | | 6.9 (13.8) | | 41.4 (27.6) | | 41.4 (34.5) | | 10.3 (58.6) | | Using feedback and reminders by professionals for improving care | | 34.5 (17.2) | 20.7 (27.6) | | 37.9 (34.5) | | 6.9 (41.4) | | Reaching agreements about the uniform use of performance indicators in the care chain | | | 3.4 (13.8) | 55.2 (27.6) | | 37.9 (37.9) | | 3.4 (51.7) | | Monitoring successes and results during the development of the integrated care chain | | | 27.6 (17.2) | | 24.1 (34.5) | | 31.0 (27.6) | | 17.2 (37.9) | | Establishing quality targets for the performance of the whole care chain | | 0 (6.9) | | 24.1 (27.6) | | 55.2 (37.9) | | 20.7 (58.6) | | Monitoring and analyzing mistakes/near mistakes in the care chain | | 6.9 (13.8) | | 6.9 (41.4) | | 62.1 (27.6) | | 24.1 (41.4) | | Using a systematic procedure for the evaluation of agreements, approaches and results | | 3.4 (6.9) | | 37.9 (20.7) | | 37.9 (48.3) | | 20.7 (51.7) | | Monitoring client judgements and satisfaction for the whole care chain | | 6.9 (10.3) | | 37.9 (27.6) | | 41.4 (44.8) | | 13.8 (51.7) | | Gathering financial performance data for the care chain | | 3.4 (10.3) | | 31.0 (27.6) | | 51.7 (31.0) | | 13.8 (55.2) | | Making transparent the effects of the collaboration on the output of the care partners | | 6.9 (10.3) | | 31.0 (37.9) | | 44.8 (37.9) | | 17.2 (48.3) | | Monitoring whether the care delivered corresponds with evidence-based guidelines | | 27.6 (13.8) | | 13.8 (48.3) | | 41.4 (17.2) | | 17.2 (34.5) | | Establishing quality targets for the performance of care partners | | 13.8 (24.1) | | 27.6 (27.6) | | 48.3 (41.4) | | 10.566778823333444232210.3 (48.3) | | Installing improvement teams at care-chain level | |
| **Cluster 4. Quality care**  This cluster contains elements that focus on the design of a multidisciplinary care pathway throughout the care chain, based on evidence-based guidelines and standards and clients’ needs and preferences. A needs assessment of the specific client group is required for this purpose, combined with the involvement of client representatives in designing, improving, and monitoring the integrated care.   | Phase 1 | **Phase 2** | **Phase 3** | **Phase 4** | **Element description** | | --- | --- | --- | --- | --- | | 31.0 (20.7) | 37.9 (44.8) | 31.0 (37.9) | 0 (51.7) | Systematically assessing the needs of the clients in the care chain | | 48.3 (20.7) | 37.9 (34.5) | 13.8 (31.0) | 0 (37.9) | Developing a multidisciplinary care pathway | | 6.9 (24.1) | 24.1 (31.0) | 55.2 (37.9) | 13.8 (55.2) | Involving client representatives in improvement projects in the care chain | | 24.1 (24.1) | 58.6 (31.0) | 13.8 (51.7) | 3.4 (51.7) | Using evidence-based guidelines and standards | | 20.7 (17.2) | 24.1 (37.9) | 37.9 (48.3) | 17.2 (44.8) | Involving client representatives by monitoring the performance of the care chain | |
| **Cluster 5. Result-focused learning**  A learning climate of striving towards continuously improved results in the care chain is this cluster’s central theme. The elements address essential ingredients for improvement: defining goals for collaboration, identifying bottlenecks and gaps in care, and ways of learning and exchanging knowledge in an open atmosphere. Incentives are used to reward improved performance.   | Phase 1 | **Phase 2** | **Phase 3** | | **Phase 4** | | **Element description** | | | --- | --- | --- | --- | --- | --- | --- | --- | | 24.1 (24.1) | 24.1 (44.8) | 27.6 (48.3) | | 24.1 (44.8) | | Stimulating a learning culture and continuous improvement in the care chain | | | 69.0 (13.8) | 13.8 (37.9) | 13.8 (31.0) | | 3.4 (37.9) | | Defining and assessing the characteristics of the collaboratively delivered care | | | 10.3 (20.7) | 34.5 (37.9) | 51.7 (27.6) | | 3.4 (37.9) | | Making transparent the benefits of the collaboration for each care-chain partner | | | 31.0 (17.2) | 27.6 (37.9) | 37.9 (41.4) | | 3.4 (48.3) | | Collaboratively assessing bottlenecks and gaps in care | | | 13.8 (34.5) | 24.1 (34.5) | 31.0 (34.5 | | 31.0 (37.9) | | Sharing knowledge among care partners about effectively organizing sustainable integrated care | | | 27.6 (34.5) | 41.4 (31.0) | 24.1 (44.8) | 6.9 (62.1) | | Striving towards an open culture for discussing possible improvements for care partners | |  | | 24.1 (24.1) | 34.5 (31.0) | 31.0 (41.4) | 10.3 (58.6) | | Learning by the exchange of information among professionals about the care process | |  | | 10.3 (20.7) | 13.8 (27.6) | 31.0 (44.8) | 44.8 (31.0) | | Integrating incentives for rewarding the achievement of quality targets | |  | | 10.3 (13.8) | 20.7 (37.9) | 51.7 (34.5) | 17.2 (62.1) | | Using knowledge and information for directing and coordinating the care chain | |  | | 10.3 (6.9) | 10.3 (37.9) | 55.2 (34.5) | 24.1 (51.7) | | Using collaborative education programs and learning environments for the professionals of care partners | |  | | 3.4 (13.8) | 6.9 (31.0) | 48.3 (31.0) | 41.4 (37.9) | | Linking consequences to the achievement of agreed goals | |  | | 6.9 (17.2) | 20.7 (31.0) | 51.7 (34.5 | 20.7 (44.8) | | Collaborative learning in the care chain in order to innovate integrated care | |  | |
| **Cluster 6. Interprofessional teamwork**  This cluster represents interprofessional teamwork for a well-defined client group. The defined client group is the target to be reached by collaborating professionals, working in well-organized multidisciplinary teams in the care chain.   | Phase 1 | **Phase 2** | **Phase 3** | **Phase 4** | **Element description** |  | | --- | --- | --- | --- | --- | --- | | 79.3 (17.2) | 17.2 (27.6) | 3.4 (24.1) | 0 (20.7) | Defining the targeted client group | | | 27.6 (13.8) | 58.6 (20.7) | 13.8 (62.1) | 0 (51.7) | Working in multidisciplinary teams |  | | 51.7 (13.8) | 31.0 (41.4) | 17.2 (48.3) | 0 (51.7) | Reaching agreements on the availability and accessibility of professionals |  | |
| **Cluster 7. Roles and tasks**  The need for clarity about each other’s expertise, roles and tasks in the care chain is reflected in this cluster. Effective collaboration at all levels, with new partners and by allocating coordinating roles are the main components.   | Phase 1 | **Phase 2** | **Phase 3** | **Phase 4** | **Element description** | | --- | --- | --- | --- | --- | | 48.3 (20.7) | 34.5 (20.7) | 13.8 (34.5) | 3.4 (34.5) | Reaching agreements among care partners on tasks, responsibilities and authorizations | | 20.7 (17.2) | 62.1 (24.1) | 17.2 (55.2) | 0 (51.7) | Achieving adjustments among care partners by means of direct contact | | 27.6 (17.2) | 51.7 (20.7) | 20.7 (41.4) | 0 ( 31.0) | Ensuring that professionals in the care chain are informed of each other’s expertise and tasks | | 41.4 (17.2) | 37.9 (6.9) | 13.8 (20.7) | 6.9 (24.1) | Installing a coordinator working at chain-care level | | 48.3 (13.8) | 31.0 (34.5) | 17.2 (27.6) | 3.4 (31.0) | Establishing the roles and tasks of multidisciplinary team members | | 10.3 (20.7) | 72.4 (13.8) | 17.2 (51.7) | 0 (51.7) | Realizing direct contact among professionals in the care chain | | 10.3 (27.6) | 6.9 (31.0) | 58.6 (17.2) | 24.1 (37.9) | Reaching agreements on introducing and integrating new partners in the care chain | | 31.0 (20.7) | 34.5 (34.5) | 27.6 (24.1) | 6.9 (34.5) | Directing the care chain by appointing a limited number of persons with coordinating tasks | |
| **Cluster 8. Commitment**  This cluster’s focus is on collaborative commitment and ambition in the care chain. Commitment towards clearly defined goals and a collaborative ambition, apart from awareness of dependencies and domains. The commitment of leaders to the care chain and the awareness of working in a care chain are also components.   | Phase 1 | **Phase 2** | **Phase 3** | **Phase 4** | **Element description** |  | | --- | --- | --- | --- | --- | --- | | 89.7 (3.4) | 0 (41.4) | 10.3 (24.1) | 0 (44.8) | Defining the ambitions and aims of the collaboration in the care chain | | | 48.3 (24.1) | 24.1 (20.7) | 17.2 (34.5) | 10.3 (34.5) | Signing collaboration agreements among care partners | | | 62.1 (20.7) | 20.7 (37.9) | 17.2 (44.8) | 6.9 (44.8) | Assuring the leadership commitment of the partners involved in the care chain | | | 51.7 (20.7) | 24.1 (31.0) | 17.2 (37.9) | 6.9 (37.9) | Describing the tasks and authorities of leaders, coordinators and advisory boards in the care chain | | | 48.3 (27.6) | 31.0 (17.2) | 17.2 (27.6) | 3.4 (34.5) | Establishing dependencies among care partners | | | 34.5 (24.1) | 34.5 (27.6) | 24.1 (44.8) | 6.9 (48.3) | Guiding the care chain by emphasizing a collaborative commitment | | | 41.4 (27.6) | 27.6 (37.9) | 20.7 (37.9) | 10.3 (51.7) | Structural meetings of leaders of care-chain organizations | | | 27.6 (37.9) | 10.3 (44.8) | 34.5 (31.0) | 27.6 (41.4) | Reaching agreements about letting go care partner domains | | | 51.7 (13.8) | 34.5 (41.4) | 10.3 (65.5) | 3.4 (58.6) | Stimulating trust among care partners | | | 31.0 (20.7) | 44.8 (37.9) | 20.7 (48.3) | 3.4 (55.2) | Stimulating the awareness of working in a care chain | | | 13.8 (20.7) | 24.1 (31.0) | 34.5 (48.3) | 27.6 (51.7) | Structural meetings with external parties such as insurers, local governments and inspectorates | | |
| **Cluster 9. Transparent entrepreneurship**  This cluster concentrates on space for innovation (experiments), leadership responsibilities for performance achievement and joint financial agreements covering the integrated care. Preconditions for entrepreneurship, including financial preconditions, are represented in the collection of elements.   | Phase 1 | | **Phase 2** | | **Phase 3** | | **Phase 4** | | **Element description** | | --- | --- | --- | --- | --- | --- | --- | --- | --- | | 58.6 (13.8) | | 6.9 (34.5) | | 24.1 (27.6) | | 10.3 (44.8) | | Making commitment to a joint responsibility for the final goals and results to be achieved | | 34.5 (24.1) | | 31.0 (37.9) | | 24.1 (44.8) | | 10.3 (51.7) | | Using a uniform language in the care chain | | 31.0 (27.6) | | 13.8 (31.0) | | 31.0 (27.6) | | 24.1 (44.8) | | Reaching agreements on the financial budget for integrated care | | 34.5 (20.7) | | 24.1 (31.0) | | 31.0 (37.9) | | 10.3 (62.1) | | Allocating financial budgets for the implementation and maintenance of integrated care | | 13.8 (27.6) | | 37.9 (27.6) | | 37.9 (37.9) | | 10.3 (48.3) | | Involving leaders in improvement efforts in the care chain | | 37.9 (10.3) | 27.6 (24.1) | | 24.1 (34.5) | | 10.3 (24.1) | | Creating an open environment that encourages experiments and pilot projects | | | 13.8 (10.3) | | 10.3 (24.1) | | 31.0 (31.0) | | 44.8 (20.7) | | Offering a single collaborative financial contract to financing parties by the collective of care partners | |
